# Benchmarking subcellular localization and variant tolerance predictors on membrane proteins

**DOI:** 10.1186/s12864-019-5865-0

**Published:** 2019-07-16

**Authors:** Tommaso Orioli, Mauno Vihinen

**Affiliations:** 1 International Master in Bioinformatics, School of Science, University of Bologna, Bologna, Italy; 2 Department of Experimental Medical Science, BMC B13, Lund University, SE-22184 Lund, Sweden

**Keywords:** Membrane protein, Benchmark, Benchmarking, Variation interpretation, Method performance, Disease-causing variant, Mutation

## Abstract

**Background:**

Membrane proteins constitute up to 30% of the human proteome. These proteins have special properties because the transmembrane segments are embedded into lipid bilayer while extramembranous parts are in different environments. Membrane proteins have several functions and are involved in numerous diseases. A large number of prediction methods have been introduced to predict protein subcellular localization as well as the tolerance or pathogenicity of amino acid substitutions.

**Results:**

We tested the performance of 22 tolerance predictors by collecting information on membrane proteins and variants in them. The analysis indicated that the best tools had similar prediction performance on transmembrane, inside and outside regions of transmembrane proteins and comparable to overall prediction performances for all types of proteins. PON-P2 had the highest performance followed by REVEL, MetaSVM and VEST3. Further, we tested with the high quality dataset also the performance of seven subcellular localization predictors on membrane proteins. We assessed separately the performance for single pass and multi pass membrane proteins. Predictions for multi pass proteins were more reliable than those for single pass proteins.

**Conclusions:**

The predictors for variant effects had better performance than subcellular localization tools. The best tolerance predictors are highly reliable. As there are large differences in the performances of tools, end-users have to be cautious in method selection.

**Electronic supplementary material:**

The online version of this article (10.1186/s12864-019-5865-0) contains supplementary material, which is available to authorized users.

## Background

Cells and compartments within them are surrounded by membranes composed of lipids having two opposed layers of amphipathic molecules. The bilayers contain in addition to lipids many other molecules, among them proteins that have numerous functions. Membrane proteins (MPs) are crucial for membrane stability and cellular functions due to their ability to communicate with the environment outside and inside of membranes. 25–30% of human proteins have been estimated to be transmembrane proteins (TMPs) [1]. These proteins are important in many ways, for example 60% of current pharmaceutical drug targets are MPs [2, 3]. Organelles within cells, such as mitochondria, endoplasmic reticulum (ER), and Golgi apparatus, are maintained by their membranes that carry specific MPs.

MPs can be classified in many ways. Structure based classification is widely used [4]. It discriminates the MPs depending on how they interact with the lipid membrane. Type I membrane proteins, also known as single pass TMPs have extracellular (or luminal) N-terminus and cytoplasmic C-terminus, while type II TMPs have the opposite: extracellular (or luminal) C-terminus and cytoplasmic N-terminus. These two types of MPs represent about half of the human membrane proteome [5]. Multi pass TMPs trespass through the membrane several times. Lipid chain-anchored membrane proteins are located on the surface of the cell membrane and are covalently attached to lipids and can be located on both sides of the membrane. Lipid attaches at or near the C-terminus of the protein and plays a crucial role in the protein function. GPI-anchored MPs are attached via glycosylphosphatidylinositol (GPI) group. Peripheral membrane proteins are bound to the membrane indirectly by non-covalent interaction with other membrane proteins. Therefore they are not considered as proper MPs.

We concentrated on TMPs of types I and II and multi pass proteins as they are permanently attached to and span through the membrane.

Besides the topology, MPs can be classified as α-helix and β-barrel proteins. α-Helical MPs are organized as anti-parallel bundles, which are typically tilted with respect to the membrane by 25° [6]. We focused on α-helical MPs, since in human, β-barrel MPs appear only in the outer membranes of mitochondria.

MPs are involved in numerous functions. Information has been collected to databases such as Orientations of Proteins in Membranes (OPM [7]), Topology Database of Transmembrane Proteins (TOPDB [8]) and Human Transmembrane Proteome (HTP [1]), in addition to more general databases including Protein Data Bank (PDB [9]) and UniProtKB [10].

In an effort to classify human membrane proteome, 6684 non-redundant genes were clustered to 234 protein families or groups, and four major functional categories [5]. Receptors mediate cellular response upon binding to a ligand. The most representative groups are G-protein coupled receptors, receptor-type kinases, receptors of immunoglobulin superfamily and scavenger receptors. Transporters move ions and molecules from the extra-cellular environment into cells, and vice versa, by utilizing electrochemical gradients or through chemical reactions. They are grouped as channels, solute carriers and active transporters. Enzymes catalyze different reactions. Miscellaneous group contains MPs that have other functions such as ligands, or structural or adhesion proteins.

The Transporter Classification Database (TCDB) is an International Union of Biochemistry and Molecular Biology (IUBMB)-approved classification system groups membrane transport proteins to seven classes: channels and pores, electrochemical potential-driven transporters, primary active transporters, group translocators, transport electron carriers, accessory factors involved in transport, and incompletely characterized transport systems [11]. According to the Enzyme Commission number classification (EC number) there are seven main classes: oxidoreductases, transferases, hydrolases, lyases, isomerases, ligases and translocases [12].

We retrieved high-quality datasets of TMPs and used them to benchmark how good protein localization predictors are since the existing benchmark studies are based on smaller and less reliable data. We collected also a dataset of disease-related variants in these TMPs and tested the performance of protein pathogenicity/tolerance predictors including the three regions in these proteins: inside and outside as well as within the membranes. This is to our knowledge the first study on the membrane protein variant prediction performance.

## Results and discussion

Our aim was to evaluate the performance of variant predictors on TMPs. For that purpose we collected datasets of both MPs and variants in them. The datasets were then used to benchmark predictors on two major characteristics of membrane proteins, namely subcellular localization and variant tolerance. The exact number of TMPs is not known, but various estimates have been presented. According to the data in HTP, the human proteome contains 25–30% of MPs. We collected information for both experimentally defined and predicted MPs.

Human Protein Atlas is a long term project for analysis of human proteins. Until now, they provide detailed experimental information for the subcellular localizations of 12,073 proteins. The data contain reliability scores. We obtained two datasets from HPA. MP1289 contains 1289 proteins reliability of which were *validated*, *supported* or *approved*.

The test sets are summarized in Table 1. In order to calculate performance indices, the negative sets were normalized to have the same number of proteins as the positive set.

**Table 1:** Numbers of proteins in test sets

	MP1289		MP508		mpHTP	
	Positive	Negative	Positive	Negative	Positive	Negative
BUSCA	1040	986	393	361	4550	4362
CELLO	1223	1180	474	449	5045	5194
DeepLoc1.0	1285	931	505	337	4669	4629
LocTree3	1286	1270	507	497	5358	5289
MultiLoc2	1106	1193	408	464	4654	4999
SubCons	852	744	309	292	4055	3882
Wolf PSORT	1289	1288	508	507	5414	5362

Additional file 1 Table S1 shows that 42% of the proteins are found in one location (542 out of 1289), the majority are multi location proteins (MLPs). Out of the 747 MLPs, 83.0% (620) were found in two locations, 16.7% (125) in three locations and 0.3% (2) in four locations. We retrieved data for 124 *validated* (9.6%), 439 *supported* (34.1%) and 726 *approved* (56.3%) proteins.

For a subset of even more reliable data we excluded those with annotation *approved* and obtained 508 proteins called MP508. The percentage of MLPs is smaller in this set, 50.6% (Additional file 1: Table S1). Out of 257 MLPs, 83.7% (215) were found in two different locations and 16.3% (42) in three different locations. The dataset contains 124 *validated* (24.4%) and 384 *supported* (75.6%) plasma membrane and nuclear MPs. The third dataset, called mpHTP, is the largest one and contains localizations of 5422 human proteins.

### Performance of subcellular localization predictors

We searched from literature and internet for protein subcellular localization predictors and found several ones. We included to our analysis seven methods that were available as web service or downloadable standalone version and allowed submission of large number of sequences. The methods are listed in Table 2. We could not test some other tools, because they did not support multiple sequence submission, including MemPype [20], HSLPred [21] and Iloc-Hum [22], or because problems with availability of the server, i.e. SherLoc2 [23]. We compared the predicted localizations to experimentally verified results and calculated six performance measures. Proteins used for training the methods were excluded when these details were available. We were not able to retrieve the training set of Wolf PSORT. Protein sequences were matched based on UniProt ID match and by similarity obtained with BLASTP [24].

**Table 2:** Subcellular localization predictors

Method	Description	URL	Reference
BUSCA	Metapredictor for localization-related protein features	http://busca.biocomp.unibo.it/	[13]
CELLO	Two-layer SVM	http://cello.life.nctu.edu.tw/	[14]
DeepLoc	Deep neural network	http://www.cbs.dtu.dk/services/DeepLoc/	[15]
LOCTREE3	SVM	https://rostlab.org/services/loctree2/	[16]
MultiLoc2	SVM	https://github.com/KohlbacherLab/MultiLoc2/tree/master/MultiLoc2	[17]
SubCons	RF	http://subcons.bioinfo.se/	[18]
Wolf PSORT	Converts amino acid sequences into numerical vectors that are grouped with a weighted k-nearest neighbor classifier	https://wolfpsort.hgc.jp/	[19]

Results in Table 1 indicate the numbers of predicted proteins for the 7 tested tools. None of the tools was able to predict all the cases in the three positive and negative test sets, however, Wolf PSORT comes very close.

Tables 3, 4 and 5 contain performance assessments for MP1289, MP508 and mpHTP, respectively. The first two datasets contain plasma membrane and nuclear membrane proteins, whereas the mpHTP contains TMPs from all sub compartments. DeepLoc1.0 and LocTree3 predict whether a protein is a MP or a cytoplasmic protein, besides the subcellular localization. Some predictors return a single result (BUSCA, DeepLoc1.0, LocTree3, SubCons) with a probability, whereas CELLO, MultiLoc2 and Wolf PSORT return the probability distribution associated to each location. In the latter case, we considered the possibility of having a double localization when there was not one with a predominant probability (i.e. > 0.50).

**Table 3:** Performance of subcellular localization predictors on MP1289

	BUSCA	CELLO	DeepLoc 1.0	LocTree3	MultiLoc2	SubCons	WolfPSORT
TP	316	348	447	344	108	242	516
FP	112	158	102	75	50	42	218
TN	763	1073	987	1203	1144	1152	1071
FN	724	875	838	942	998	610	773
Sensitivity	0.36	0.28	0.41	0.27	0.09	0.2	0.4
Specificity	0.87	0.87	0.91	0.94	0.96	0.96	0.83
PPV	0.74	0.69	0.81	0.82	0.68	0.85	0.7
NPV	0.51	0.55	0.54	0.56	0.53	0.65	0.58
ACC	0.56	0.58	0.6	0.6	0.54	0.68	0.62
MCC	0.21	0.19	0.3	0.28	0.11	0.35	0.26
OPM	0.23	0.21	0.28	0.26	0.18	0.3	0.25

**Table 4:** Performance of subcellular localization predictors on MP508

	BUSCA	CELLO	DeepLoc1.0	LocTree3	MultiLoc2	SubCons	Wolf PSORT
TP	126	136	218	166	49	110	227
FP	18	28	20	10	9	6	40
TN	282	438	350	493	437	440	468
FN	267	338	287	341	359	199	281
Sensitivity	0.42	0.29	0.59	0.33	0.11	0.25	0.45
Specificity	0.94	0.94	0.95	0.98	0.98	0.99	0.92
PPV	0.88	0.83	0.92	0.94	0.84	0.95	0.85
NPV	0.51	0.56	0.55	0.59	0.55	0.69	0.62
ACC	0.59	0.61	0.65	0.65	0.57	0.73	0.68
MCC	0.32	0.30	0.42	0.41	0.20	0.47	0.42
OPM	0.29	0.27	0.38	0.34	0.22	0.37	0.35

**Table 5:** Performance of subcellular localization predictors on mpHTP

	BUSCA	CELLO	DeepLoc1.0	LocTree3	MultiLoc2	SubCons	Wolf PSORT
TP	3766	3101	4242	3877	1187	2776	4016
FP	206	153	257	179	104	103	280
TN	4156	5041	4372	5110	4895	3779	5082
FN	784	1944	427	1481	3467	1279	1398
Sensitivity	0.86	0.60	0.93	0.73	0.24	0.71	0.75
Specificity	0.95	0.97	0.94	0.97	0.98	0.97	0.95
PPV	0.95	0.95	0.94	0.96	0.92	0.96	0.93
NPV	0.84	0.72	0.91	0.78	0.58	0.75	0.78
ACC	0.89	0.79	0.93	0.84	0.63	0.83	0.84
MCC	0.78	0.63	0.85	0.71	0.34	0.68	0.70
OPM	0.72	0.53	0.80	0.62	0.30	0.60	0.62

The performance of all methods is very low on both MP1289 and MP508 (Tables 3 and 4). The MCC values range from 0.11 (MultiLoc2) to 0.35 (SubCons) in the MP1289 and from 0.20 (MultiLoc2) to 0.47 (SubCons) for MP508. In addition to having low overall performance, the tools are very biased, especially sensitivity is very low while specificity has the highest score among the tested ones for all the methods and on both the datasets.

All the measures are somewhat better for MP508. The tools are largely under predicting MPs. When just about one out of three or four of the real MPs are predicted correct, the overall performance remains low (Tables 3 and 4). Consequently, the number of false negatives is very high. Specificity is higher, but that is because the number of false negatives is two times higher than that for the true positives. It can be concluded that if these tools predict a protein to be an MP it is highly likely true, the problem is that they miss 60% or more of the cases.

The performance scores are better for the mpHTP set. The MCC ranges from 0.85 for DeepLoc1.0 to 0.34 for MutliLoc2. In this case also the sensitivity is clearly better, from 0.60 to 0.93, except for MultiLoc2, which has a value of 0.24. DeepLoc1.0 has the best and MultiLoc2 the lowest OPM on all the datasets. The performances are contradictory for the other methods, each of BUSCA, LocTree, SubCons and Wolf PSORT showing good performance on some datasets. BUSCA, DeepLoc and LocTree3 can predict either the subcellular localization or whether a protein is a MP. On the mpHTP data, DeepLoc1.0 is clearly the best balanced method, since values for sensitivity, specificity, PPV and NPV span from 0.91 to 0.94.

As seen above, the results are very sensitive for the composition of the dataset. The majority of the proteins in MP1289 and MP508 are MLPs, 57.7 and 50.6% respectively. When we filtered out all the MLPs from the MP1289 and reassessed the performance, the performances increased, but still the best performing tools (DeepLoc1.0 and Wolf PSORT) had MCC of 0.51 and OPM of 0.43 and 0.42 (Additional file 1: Table S2).

Next, we evaluated the performance of the predictors by dividing the mpHTP dataset into two subsets, those containing one TM region i.e. single pass proteins and those containing > 1 TM region (multi pass). The results indicate that all the predictors are performing better for multi pass proteins (Additional file 1: Table S3). Now, in addition to BUSCA and DeepLoc1.0, CELLO excels with second highest MCC of 0.88. One explanation for the better detection of multi pass proteins may be that since there are several TM regions the predictors have more chances in detecting them.

### Performance of tolerance predictors on variants in MPs

Tolerance predictors are widely used to investigate outcomes of identified variants. Tens of such methods have been developed. Performances of these methods have been previously tested [25–28], but not specifically for MPs. As the training datasets of machine learning methods (ML) do not contain that many MPs, it was of interest to find how the tools work on membrane proteins. We collected a set of 2058 variants, 747 of which were disease-related and 1311 which had high (> 1% but < 25%) minor allele frequency in ExAC and which can be considered as benign.

We tested altogether 22 methods, principles of which are widely different, for a summary see [29]. The methods were run on default parameters. Variants used to train PON-P2 (7016 in TMPs, 3990 deleterious and 3026 neutral) were excluded from tests.

**Fig. 1:**
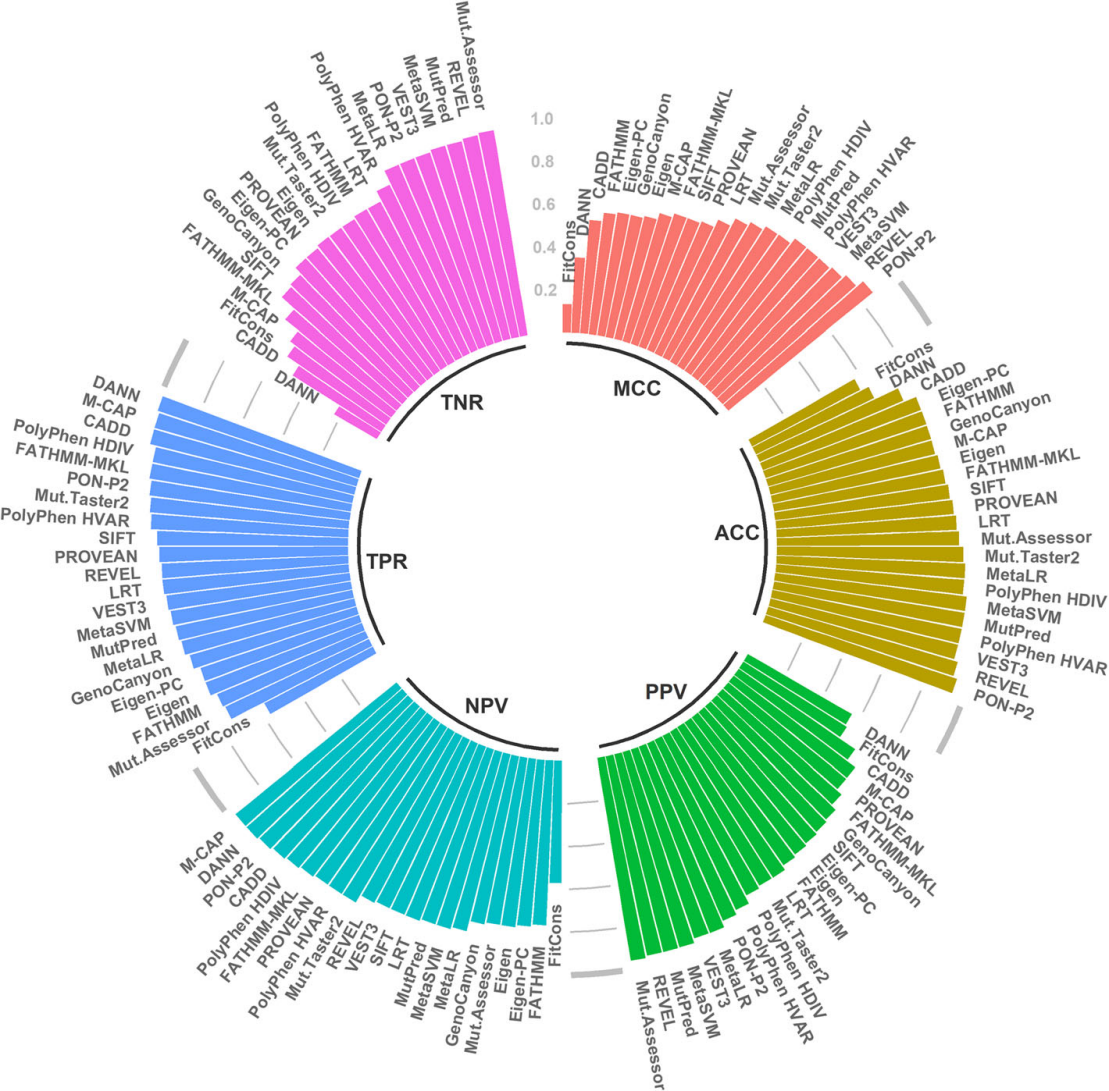
Visualisation of six performance measures for tolerance predictors. The methods are organized according to their increasing performance for each of the measures

The general performances of all the predictors are summarized in Table 6 and in Fig. 1. Similar to previous assessments on proteins in general, the performances vary widely. The MCC values range from 0.13 for fitCons to 0.87 for PON-P2 and the OPM from 0.18 (fitCons) to 0.82 (PON-P2). Altogether, six methods have the MCC equal or higher than 0.80, namely PON-P2 (0.87), REVEL (0.83), MetaSVM (0.81), MutPred (0.80), PolyPhen HVAR (0.80) and VEST3 (0.80).

**Table 6:** Overall performance of tolerance predictors on MP variants

	TP	FP	TN	FN	Sensitivity	Specificity	PPV	NPV	ACC	MCC	OPM
CADD	725	670	641	18	0.98	0.49	0.66	0.95	0.73	0.53	0.44
DANN	737	1006	305	6	0.99	0.23	0.56	0.97	0.61	0.35	0.30
Eigen	582	253	1058	161	0.78	0.81	0.80	0.79	0.80	0.59	0.50
Eigen-PC	580	266	1045	163	0.78	0.80	0.79	0.78	0.79	0.58	0.49
FATHMM	556	230	1053	183	0.75	0.82	0.81	0.77	0.79	0.57	0.49
FATHMM-MKL	705	434	877	38	0.95	0.67	0.74	0.93	0.81	0.64	0.55
FitCons	423	571	740	320	0.57	0.56	0.57	0.57	0.57	0.13	0.18
GenoCanyon	605	313	998	138	0.81	0.76	0.77	0.80	0.79	0.58	0.49
LRT	625	205	904	91	0.87	0.82	0.83	0.87	0.84	0.69	0.60
M-CAP	707	104	152	13	0.98	0.59	0.71	0.97	0.79	0.62	0.53
MetaLR	615	76	1235	128	0.83	0.94	0.93	0.85	0.88	0.77	0.70
MetaSVM	629	59	1252	114	0.85	0.95	0.95	0.86	0.90	0.81	0.74
MutationAssessor	278	36	999	96	0.74	0.97	0.96	0.79	0.85	0.73	0.64
MutationTaster2	694	251	1060	49	0.93	0.81	0.83	0.92	0.87	0.75	0.67
MutPred	624	62	1240	117	0.84	0.95	0.95	0.86	0.90	0.80	0.73
PolyPhen HDIV	668	213	925	37	0.95	0.81	0.84	0.94	0.88	0.77	0.69
PolyPhen HVAR	633	151	1046	53	0.92	0.87	0.88	0.92	0.90	0.80	0.73
PON-P2	699	76	1235	48	0.94	0.94	0.90	0.96	0.94	0.87	0.82
PROVEAN	646	263	1040	92	0.88	0.80	0.71	0.92	0.83	0.65	0.56
REVEL	650	56	1255	93	0.87	0.96	0.95	0.88	0.92	0.83	0.77
SIFT	659	330	979	84	0.89	0.75	0.78	0.87	0.82	0.64	0.55
VEST3	641	79	1232	102	0.86	0.94	0.93	0.87	0.90	0.80	0.73

Out of 2058 variants in the dataset, 1934 (94%) were correctly predicted by the best performing tool (PON-P2): 338 out of 367 (92.1%) in transmembrane region and 1559 out of 1691 (92.2%) in non-transmembrane region. Of 747 pathogenic and 1311 neutral variants, 699 (93.6%) and 1235 (94.2%) were correctly predicted, respectively.

**Fig. 2:**
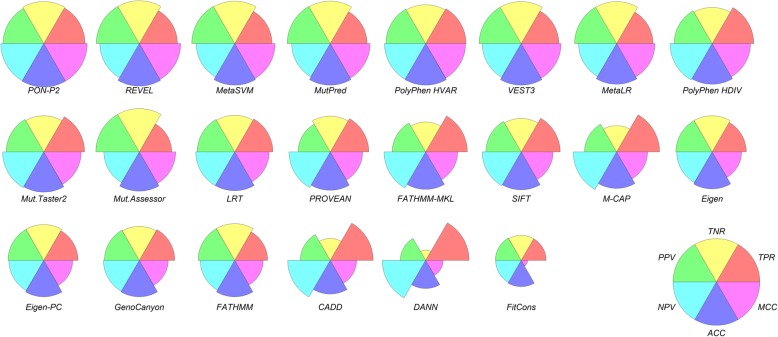
Visualization of the performance of tolerance predictors for MP variants. The graphs indicate the performance of each measure as well as how balanced the methods are. Good predictors are balanced and predict both positive and negative cases equally well

**Fig. 3:**
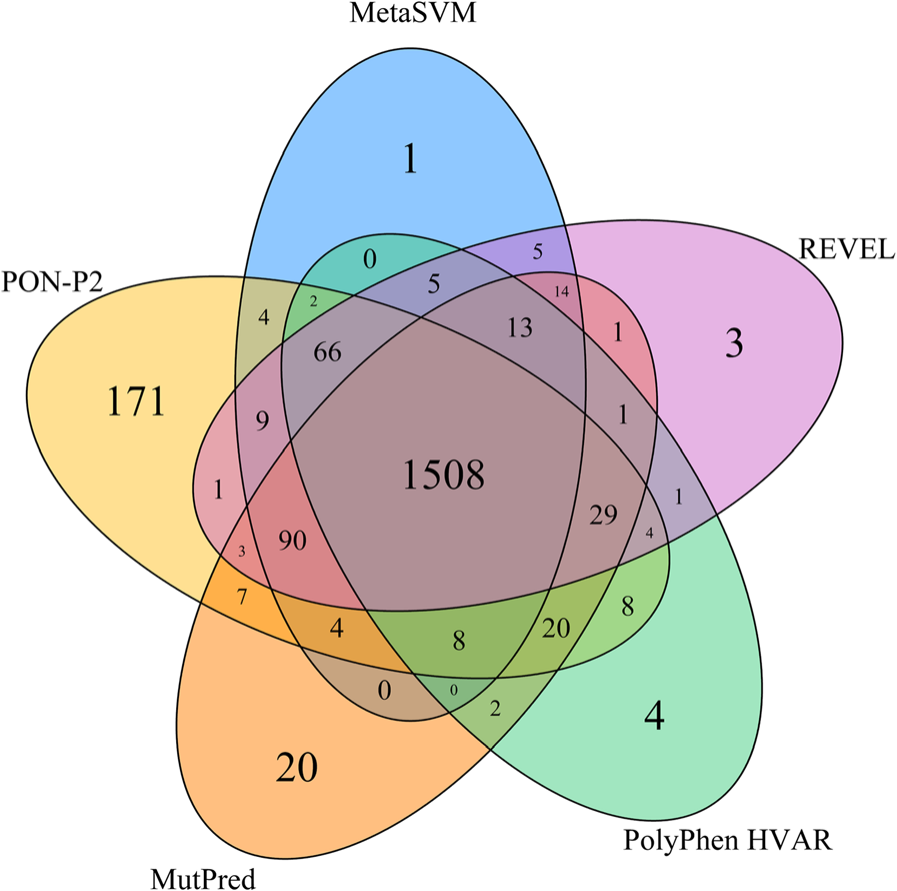
Venn diagram for the congruence of the five best performing tools. The results are shown for 2002 variants that all the tools predicted. All the five methods predicted correct 1508 (75.3%) variants

There are no major differences for the three regions in the TMPs, i.e. transmembrane, the inner region and the outer region (Table 7), except for Eigen, Eigen-PC, fitCons, and MutationAssessor when looking at accuracy, MCC and OPM. Twelve of the methods have the best performance for inner membrane variants, 5 methods for outer membrane and 5 methods for transmembrane regions, however the differences are typically very small. Of the 10 best methods, seven perform somewhat better for outer membrane and 3 for transmembrane variants than on the other parts in the MPs. The best methods, in particular PON-P2 and REVEL, are also the most balanced when all the assessment measures are considered. Methods with lower performances, such as CADD, DANN and fitCons are the most unbalanced (Fig. 2). Venn diagram in Fig. 3 shows the numbers of correct predictions by the five best performing tools. The superior performance of PON-P2 originates from its capability alone or together with MetaSVM or MutPred to predict correct cases beyond what all the methods agree on.

**Table 7:** Performance of tolerance predictors divided to membrane protein parts

	Sensitivity			Specificity			PPV			NPV			ACC			MCC			OPM		
	TM^a^	I^a^	O^a^	TM	I	O	TM	I	O	TM	I	O	TM	I	O	TM	I	O	TM	I	O
CADD	0.99	0.96	0.98	0.46	0.50	0.49	0.65	0.66	0.66	0.99	0.93	0.96	0.73	0.73	0.73	0.54	0.52	0.54	0.45	0.43	0.45
DANN	1.00	0.99	0.99	0.20	0.24	0.24	0.56	0.57	0.56	1.00	0.97	0.95	0.60	0.62	0.61	0.33	0.35	0.34	0.30	0.31	0.30
Eigen	0.78	0.89	0.70	0.73	0.83	0.82	0.74	0.84	0.79	0.77	0.88	0.73	0.76	0.86	0.76	0.51	0.71	0.52	0.43	0.63	0.44
Eigen-PC	0.78	0.88	0.70	0.74	0.80	0.81	0.75	0.82	0.79	0.77	0.87	0.73	0.76	0.84	0.76	0.52	0.69	0.51	0.44	0.60	0.43
FATHMM	0.63	0.76	0.81	0.86	0.78	0.84	0.82	0.77	0.84	0.70	0.76	0.82	0.75	0.77	0.83	0.51	0.54	0.66	0.43	0.45	0.57
FATHMM-MKL	0.97	0.93	0.96	0.63	0.67	0.68	0.73	0.74	0.75	0.95	0.90	0.94	0.80	0.80	0.82	0.64	0.62	0.66	0.54	0.53	0.57
FitCons	0.44	0.69	0.55	0.72	0.55	0.53	0.61	0.60	0.54	0.56	0.64	0.54	0.58	0.62	0.54	0.17	0.24	0.08	0.20	0.24	0.16
GenoCanyon	0.78	0.84	0.81	0.72	0.77	0.77	0.74	0.78	0.78	0.76	0.83	0.80	0.75	0.81	0.79	0.50	0.61	0.58	0.42	0.52	0.49
LRT	0.94	0.85	0.85	0.75	0.82	0.83	0.79	0.83	0.83	0.93	0.85	0.85	0.85	0.84	0.84	0.70	0.67	0.68	0.62	0.59	0.59
M-CAP	0.99	0.98	0.98	0.55	0.57	0.64	0.69	0.69	0.73	0.98	0.96	0.97	0.77	0.77	0.81	0.60	0.60	0.66	0.50	0.50	0.56
MetaLR	0.81	0.81	0.85	0.92	0.93	0.96	0.91	0.92	0.95	0.83	0.83	0.87	0.86	0.87	0.91	0.73	0.74	0.82	0.65	0.66	0.75
MetaSVM	0.85	0.83	0.86	0.93	0.95	0.97	0.92	0.94	0.97	0.86	0.85	0.87	0.89	0.89	0.91	0.78	0.78	0.83	0.70	0.71	0.77
MutationAssessor	0.89	0.60	0.78	0.92	0.98	0.97	0.91	0.97	0.96	0.89	0.71	0.82	0.90	0.79	0.88	0.80	0.63	0.77	0.73	0.53	0.69
MutationTaster2	0.99	0.92	0.91	0.78	0.81	0.82	0.82	0.83	0.83	0.99	0.91	0.90	0.89	0.87	0.86	0.79	0.74	0.73	0.71	0.65	0.65
MutPred	0.87	0.78	0.88	0.91	0.97	0.95	0.91	0.96	0.95	0.87	0.81	0.89	0.89	0.87	0.92	0.78	0.76	0.83	0.70	0.68	0.77
PolyPhen HDIV	0.96	0.94	0.94	0.70	0.83	0.83	0.76	0.85	0.85	0.95	0.93	0.94	0.83	0.89	0.89	0.69	0.78	0.78	0.59	0.70	0.70
PolyPhen HVAR	0.95	0.90	0.92	0.75	0.90	0.89	0.80	0.90	0.89	0.94	0.90	0.92	0.85	0.90	0.91	0.72	0.80	0.81	0.63	0.73	0.74
PON-P2	0.94	0.93	0.94	0.90	0.94	0.96	0.90	0.88	0.92	0.94	0.96	0.97	0.92	0.93	0.95	0.85	0.85	0.89	0.78	0.80	0.85
PROVEAN	0.91	0.83	0.89	0.70	0.81	0.81	0.75	0.82	0.83	0.89	0.83	0.88	0.81	0.82	0.85	0.63	0.65	0.71	0.54	0.56	0.62
REVEL	0.90	0.85	0.88	0.93	0.96	0.97	0.93	0.95	0.96	0.91	0.86	0.89	0.92	0.90	0.92	0.84	0.81	0.85	0.77	0.74	0.79
SIFT	0.94	0.86	0.88	0.65	0.74	0.78	0.73	0.77	0.80	0.91	0.84	0.87	0.80	0.80	0.83	0.62	0.60	0.67	0.52	0.52	0.58
VEST3	0.95	0.86	0.82	0.90	0.94	0.95	0.90	0.94	0.94	0.95	0.87	0.84	0.92	0.90	0.88	0.85	0.81	0.77	0.79	0.74	0.70

^a^I, inside the membrane; O, outside the membrane; TM transmembrane

The observed performance scores for the tools are close to the measures obtained when benchmarking with all kinds of proteins [25–28, 30]. Although the ratio of membrane protein variants has been small during the training of many of the predictors, it does not show in the performances. This could be because despite MPs are embedded into lipid bilayers, only the surface of the transmembrane regions in MPs is in contact with lipids, for the other residues the environment is similar to internal positions in proteins or in protein complexes in general.

### Estimation of the sensitivity of MPs for variations

Recently we estimated the sensitivity of nine groups of proteins for harmful variants based on PON-P2 predictions [31]. PON-P2 was found to have the best performance also for all the three regions in MPs (Table 6). As the results for all possible variants for this method were not available, we used a script to submit all 19 variants in every position in the MPs to the program in batches.

We created all the variants of the entire mpHTP (5422 proteins), a total of 56,686,557 variants in 2,983,503 amino acids. The results are summarized in Table 8. In addition to overall sensitivity, we investigated whether the different regions of the TMPs had different sensitivities for variations. The variants were classified in three classes: neutral, pathogenic and those of unknown significance. 48.0% of the predicted positions in MPs were outside the membrane, 15.3% transmembrane, and 36.7% inside the membrane.

**Table 8:** Statistics for predicted variants in human membrane proteome

	Total	Outer	Transmembrane	Inner
Number of proteins	5422	–	–	–
Number of predicted proteins	5070	–	–	–
Predicted proteins (%)	93.51	–	–	–
Number of amino acids	2,983,503	1,458,196	456,186	1,069,121
Number of predicted amino acids	2,850,519	1,367,843	434,745	1,047,931
Predicted amino acids (%)	95.54	93.80	95.30	98.02
Number of possible variants in all proteins/region	56,686,557	27,705,724	8,667,534	20,313,299
Number of possible variants in predicted proteins/region	54,159,861	25,989,017	8,260,155	19,910,689
Number of predicted variants	53,310,412	25,558,804	8,169,606	19,581,983
Predicted variants (% of possible)	98.43	98.34	98.90	98.35
Number of variants predicted as neutral	21,343,305	10,529,555	3,141,354	7,672,377
Neutral variants (%)	40.04	41.20	38.45	39.18
Average number of neutral variants per protein	4197.31	2070.71	617.77	1508.83
Median number of neutral variants per protein	2471	727	234	781
Number of variants predicted as pathogenic	9,760,571	4367,037	1,702,467	3,691,067
Pathogenic variants (%)	18.31	17.09	20.84	18.8
Average number of pathogenic variants per protein	1919.48	858.81	334.80	725.87
Median number of pathogenic variants per protein	173	19	2	20
Number of variants predicted as unknown	22,206,536	10,662,212	3,325,785	8,218,539
Unknown variants (%)	41.66	41.72	40.71	41.97
Average number of unknown variants per protein	4367.07	2096.80	654.04	1616.23
Median number of unknown variants per protein	3013	714	290	616
Ratio of pathogenic and neutral variants	0.46	0.41	0.54	0.48

We obtained results for 5070 proteins (93.5% of mpHTP entries) with high prediction coverage (95.5%). There are several reasons for not reaching full coverage. Some proteins are unique for human, thus the required evolutionary information is missing and the predictor cannot work. Some sequences contained special characters, possibly for residue ambiguity. Those were not included to the analysis. Variations inside the membranes are predicted with slightly higher rate than transmembrane or outside the membrane regions. The rate of unknown classifications is 41.66% and it is almost invariant for the three regions. This category contains many types of variants. The ratio is similar to many other proteins [31]. This category reflects also heterogeneity and continuum nature of pathogenicity [32]. The results are rather even for all the three regions in MPs. Transmembrane regions contain somewhat more likely pathogenic variants than the extramembranous regions: the ratio is 0.54 vs 0.41 and 0.48. The share of neutral variants varies between 38.45 to 41.20%. Totally close to 10 million variations were predicted to be pathogenic.

## Conclusion

We tested the performance of two types of predictors on membrane proteins, their subcellular localization and variation interpretation in these proteins. We tested 22 variant tolerance predictors and 7 subcellular localization tools. The best variation prediction methods had similar prediction performance on transmembrane, inside and outside regions of transmembrane proteins and comparable to overall prediction performances for all types of proteins. The highest performing method was PON-P2, followed by REVEL, MetaSVM and VEST3. In the case of subcellular localization predictors we assessed separately the performance for single pass and multi pass membrane proteins. Predictions for multi pass proteins were more reliable than those for single pass proteins. Finally, we predicted the effects of all possible 19 substitutions in 5422 membrane proteins, a total of 56,686,557 variants in 2,983,503 residues. Transmembrane regions seem to be somewhat more vulnerable for variations that regions inside and outside the membranes.

## Methods

### Data for transmembrane proteins

TMPs were obtained from two resources - Human Protein Atlas (HPA) and Human Transmembrane Proteome.

HPA [33–35] is a human proteome database based on quantitative transcriptomic analyses on tissue and organ levels. All major tissues and organs (*n* = 44) were analyzed by using 20,456 proprietary and 3572 externally obtained antibodies, totally providing more than 13 million immunochemistry images.

HPA database is organized in three main sections to tissue, cell and pathology atlases. Protein localization information was retrieved from the Cell Atlas section, where there are data for the subcellular localization of 12,073 proteins. The proteins are classified into 13 major organelles: actin filaments, centrosome, microtubules, intermediate filaments, cytosol, mitochondria, plasma membrane, vesicles, ER, Golgi apparatus, nuclear membrane, nucleoli, or nucleus. When using data from HPA, we considered as MPs proteins located to plasma membrane (including cell junctions) or nuclear membrane.

The reliability of data in HPA is indicated with four labels [34]. Location is *validated*, when it is according to one of the validation “pillars” proposed by an international working group [36]. Location is *supported*, when there is an agreement with the external experimental data from UniProtKB database. Location is *approved*, when external experimental information about the protein location is lacking, and location is *uncertain*, when there is contradictory information, such as with literature or transcriptomics data. HPA contains subcellular location data for 12,073 proteins [34].

HTP [1] is a database that combines experimental topology data together with predictions of the human transmembrane proteome. HTP is based on UniProt (UniRef 90) Human Proteome of 19,584 proteins. The proteins were filtered by using the Constrained Consensus Topology prediction method (CCTOP) [37] by using a consensus of ten major topology prediction methods. The current number of experimental and predicted MPs is 5423 (last updated August 7, 1917). The database contains topology information for each of the proteins.

### MP datasets

Three datasets of TMPs were built to test predictors. They are available in VariBench [38] at [39].

#### MP1289

TMPs were filtered from the HPA location data as follows: the main location had to be one of the following: plasma membrane, cell junctions or nuclear membrane; the annotation for reliability score was *validated*, *supported* or *approved*; and the immunofluorescence (IF) location score of the three selected locations (plasma membrane, cell junction, nuclear membrane) was *validated*, *supported* or *approved*. Note that many proteins are localized to several compartments. The filtering yielded 1289 MPs. The sequences of these proteins were retrieved in fasta format from UniProt [10].

A negative set with the same number of proteins was obtained by picking randomly 1289 non-MP proteins by filtering out plasma membrane, cell-junction and nuclear membrane proteins from Cell Atlas. Since many MPs reside in several cellular organelles, we filtered out also proteins labelled to mitochondrion, ER, Golgi apparatus or vesicles. Further, we retrieved the “Subcellular location” information from UniProt for the proteins and filtered out proteins with membrane locations such as “cell membrane”, “single-pass type II membrane protein” or “multi-pass membrane protein”.

#### MP508

To obtain even more reliable experimentally validated dataset we excluded proteins annotated as *approved* from the MP1289 dataset and obtained 508 *validated* or *supported* proteins. As for the MP1289, a negative set was obtained by randomly choosing 508 non-MP proteins from Cell Atlas, excluding organelle proteins.

#### mpHTP

Since HPA database does not contain protein topology information, we obtained proteins with these details from HTP database. It contained topology information, experimental or predicted by very reliable CCTOP algorithm [37]. The topology is defined as I, M, O, L, S, T and U labels corresponding to cytoplasmic loops, membrane spanning segments, non-cytoplasmic loops, membrane re-entrant loops, signal peptides, transit peptides, and unknown regions, respectively [1]. In the entire mpHTP dataset of 5423 proteins, we found that two entries (HTP_152398 and HTP_010883) referred to the same protein with UniProt code P0DM63. Thus, the final number of entries in the mpHTP was 5422. By comparing the UniProt codes with DS1289 and DS508, we found that 450 and 205 proteins, respectively, were annotated in mpHTP.

We retrieved a negative dataset by downloading non-TMPs from HTP and randomly selecting the same number as in the positive set (5422).

### Dataset for variants in MPs

We could not find dedicated datasets for variants in MPs. Data from MutHTP [40] could not be used as many of the included cases are from cancers and without any evidence for disease. Therefore we collected a new dataset and mapped the locations of variants within the proteins. We used several sources. Non-disease related variants were obtained from Exome Aggregation Consortium (ExAC) dataset [41] among variants with a frequency higher than 1% but lower than 25% at least in one population [28]. Disease related membrane protein variants used to train PON-P2 [30] were obtained from VariBench. Additional cases were identified by searching locus specific variation databases (LSDBs) from LOVD for MPs identified in the first step. Additional databases were identified from Mutation Update articles in Human Mutation.

By combining and pruning the identified variants for duplicates and e.g. in proteins with *unknown* reliability score in HPA, we retrieved totally 2058 variants (MPvar). There were 747 disease-related and 1311 benign cases.

### Subcellular localization prediction methods

Subcellular localization prediction methods that allowed submission of large numbers of sequences were identified with literature and internet searches. The available and tested methods are described below.

#### BUSCA

The Bologna Unified Subcellular Component Annotator (BUSCA) [13] integrates prediction tools developed at the Bologna Biocomputing Group. It contains two types of predictors. There are methods for particular subsequence regions related to subcellular location, such as signal and transit peptides, GPI anchors and transmembrane domains. These methods include DeepSig [42], TPred3 [43], PredGPI [44], ENSEMBLE3.0 [45] and BetAware [46]. The second category includes methods predicting subcellular localization directly and includes BaCelLo [47], SChloro [48] and MemLoci [49].

They used two training subsets, Critical Assessment of Function Annotation 2 (CAFA2) data containing 2512 proteins from animals, 26 from fungi, 105 from plants, 87 from Gram-negative and 2 from Gram-positive bacteria; and CAFA3 data for 2559 proteins from animals, 535 from fungi, 489 from plants, 165 from Gram-negative and 16 from Gram-positive bacteria. The web server admits a maximum of 500 fasta sequences of up to 400,000 residues.

#### Cello

The subCELlular LOcalization (CELLO) predictor [14] is a multi-class support vector machine (SVM) classifier that uses a two-level system. In the first layer, the protein sequence is decomposed to extract four types of sequence coding schemes: amino acid composition, di-peptide composition, partitioned amino acid composition, and sequence composition based on the physicochemical properties of amino acids. Four independent SVM predictors were trained independently to generate probability distributions of the subcellular localizations. In the second layer the four schemes are combined to generate the final probability distribution of subcellular compartments and the localization with the highest probability.

For training, two datasets were used: 1444 proteins from Gram-negative bacteria distributed in five different subcellular compartments (extracellular, cytoplasmic, cytoplasmic membrane, periplasmic, outer membrane), and 7589 eukaryotic proteins distributed in 12 subcellular localizations (chloroplast, cytoplasmic, cytoskeleton, ER, extracellular, Golgi apparatus, lysosomal, mitochondrial, nuclear, peroxisomal, plasma membrane, vacuolar). Based on the input sequence and the cell type, CELLO returns a probability distribution of the subcellular localization and highlights the most probable one(s).

#### DeepLoc1.0

DeepLoc1.0 [15] is a deep learning predictor of recurrent neural networks (RNNs) with long-short-term memory (LSTM) cells, attention models and convolutional neural networks (CNNs). The CNNs extract short motifs from the sequence using 120 filters (20 for each of the sizes 1, 3, 5, 9, 15 and 21 residues). The RNN scans the sequence in both directions using 256 LSTM units and has totally 1,000,512 dimensional output. The attention decoding layer uses an LSTM with 512 units through 10 decoding steps while the attention mechanism feedforward neural network (FFN) has 256 units. The final fully connected layer has one unit for membrane-bound and 10 units for other subcellular localizations [15].

DeepLoc training data was from UniProt and contained 13,858 proteins in 10 subcellular localizations (nucleus, cytoplasm, extracellular, mitochondrion, cell membrane, ER, plastid, Golgi apparatus, lysosome/vacuole, peroxisome). The web-server allows two types of predictions: accurate, for maximum of 50 sequences, and fast, for a maximum of 500 sequences.

#### LocTree3

LocTree3 [16] is an SVM approach to predict 18 eukaryotic subcellular localizations, 6 for bacteria and in 3 for Archaea. The SVMs are combined with homology-based inference by transferring localization annotations by homology through PSI-BLAST [50].

The training dataset contained three sub-datasets: 1682 eukaryotic proteins with 18 locations, 479 bacterial proteins with 6 locations, and 53 archaeal proteins with 3 locations. For each training set a different SVM was trained to build specific predictors. Web server and a standalone version are both available. We used the web server as it had no limitations for the number of submitted sequences.

#### MultiLoc2

MultiLoc2 [17] utilizes SVM predictor MultiLoc (Höglund et al. 2006), based on overall amino acids and the presence of known sorting signals, combined with phylogenetic profiles and Gene Ontology (GO) terms. MultiLoc2 integrates six subclassifiers: SVMTarget predicts localization categories based on N-terminal targeting sequences; SVMaac predicts localization based on overall amino acids composition; SVMSA predicts localization based on the presence of signal anchors; MotifSearch prediction is based on particular sequence motifs and structural domains; PhyloLoc utilizes information from homologous proteins in 78 fully sequenced genomes; and GOLoc takes benefit of GO annotations.

HighRes mode predicts 11 subcellular localizations (nuclear, cytoplasmic, mitochondrial, chloroplast, extracellular, plasma membrane, peroxisomal, ER, Golgi apparatus, lysosomal, vacuolar), whereas the LowRes is specialized for globular proteins and predicts 4 subcellular localizations (nuclear, cytoplasmic, mitochondrial, chloroplast). We used the HighRes mode.

MultiLoc2 training set contained 5959 sequences divided into 11 subcellular localizations. As the MultiLoc2 web server allows only 20 sequences per submission, we used a standalone version.

#### SubCons

SubCons [18] is based on ensemble approach, which combines the prediction results of CELLO2.5, LocTree2, MultiLoc2 and SherLoc2 using a Random Forest (RF) classifier. It classifies proteins into nine compartments (nucleus, cytoplasm/cytoskeleton, mitochondria, peroxisome, ER, Golgi apparatus, lysosome, plasma membrane, extracellular/secreted). SubCons returns a single subcellular localization result.

The training set contained 5484 proteins annotated in at least one experimental study. The SubCons web server has no sequence limit, so we used it. There is also a standalone version available.

#### Wolf PSORT

Wolf PSORT [19] is an extension of PSORT II [51] and uses PSORT [52] localization features to predict some features from iPSORT [53] along with amino acid composition. The features are used to convert amino acid sequences into numerical vectors, which are then classified with a weighted k-nearest neighbor classifier. Wolf PSORT classifies proteins into more than 10 localizations, including dual ones.

The training set was divided into fungi, plant and animal data containing 2113, 2333 and 12,771 proteins, respectively. The web server has no sequence input limits. Wolf PSORT returns the most probable localization with a number that roughly indicates the number of nearest neighbors to the query which localize to each site adjusted to account the possibility of dual localization.

### Variant tolerance predictors

Large numbers of tools have been released for the prediction of pathogenicity or tolerance of amino acid substitutions. We tested the performance of 22 variant predictors on MP variants.

The methods included Combined Annotation Dependent Depletion (CADD) [54], Deleterious Annotation of genetic variants using Neural Networks (DANN) [55], Eigen [56], Eigen-PC [56], Functional Analysis through Hidden Markov Models (FATHMM) [57], FATHMM-MKL [58], fitCons [59], GenoCanyon [60], Likelihood Ratio Test (LRT) [61], Mendelian Clinically Applicable Pathogenicity (M-CAP) [62], MetaLR [63], MetaSVM [63], MutationAssessor [64], MutationTaster2 [65], MutPred [66], Polymorphism Phenotyping v2 (PolyPhen) HDIV [67], PolyPhen HVAR [67], PON-P2 [30], Protein Variation Effect Analyzer (PROVEAN) [68], Rare Exome Variant Ensemble Learner (REVEL) [69], Sorting Intolerant From Tolerant (SIFT) [70], and Variant Effect Scoring Tool (VEST3) [71]. Variant effect predictions were downloaded from dbNSFP [72] apart for PON-P2, which were submitted via the program web site.

The tools can be classified based on how they have been implemented. Sequence information is the only feature in LRT, PROVEAN, and SIFT. These methods generate scoring data for sequence positions based on multiple sequence alignments of related sequences. Machine learning methods utilize various types of features for conservation, sequence characteristics, information about protein functions, propensities of the original and variant amino acids etc. Machine learning methods include CADD, DANN, Eigen, Eigen-PC, FATHMM, FATHHMM-MKL, GenoCanyon, M-CAP, MetaLR, MetaSVM, MutationAssessor, MutationTaster2, MutPred, PolyPhen both with HDIV and HVAR data, PON-P2, REVEL, and VEST. The machine learning-based methods are either unsupervised or supervised. The supervised methods have been trained on known disease-causing and benign variants. Algorithms in these tools include Bayesian, Hidden Markov Model (HMM), neural network (NN), RF, SVM and other approaches. fitCons is based on clustering of functional genomic fingerprints and fitness calculations.

CADD ranks single nucleotide variants (SNVs) and short insertions and deletions. It assumes two types of variants: the *proxy-neutral* variants, fixed by purifying selection, and *proxy-deleterious* variants, from de novo variations free of positive selection. DANN is a deep neural network approach trained with about 30 millions of variants. It is very close to CADD.

Eigen is an unsupervised spectral approach. Its main assumption is that the variants can be partioned in two distinct groups: functional and non-functional. A weighted linear combination of annotations is constructed, based on these estimated accuracies. Eigen-PC is conceptually simpler, based on eigen decomposition of the annotation covariance matrix. It uses the lead eigenvector to weight the individual annotations.

FATHMM is species-independent but with optional species-specific weightings. It creates an Hidden Markov Model based multiple sequence alignment and protein domain analysis. It derives weights from the relative frequencies of disease-associated and functionally neutral amino acid substitutions mapped onto conserved protein domains. FATHMM-MKL is a SVM tool that in addition to substitutions predicted effects of insertions and deletions.

fitCons estimates variation fitness consequences according to functional genomic fingerprints by integrating evolutionary and functional data.GenoCanyon is an unsupervised statistical learning method. It provides a posteriori probabilities for functional genomic positions and are used as deleteriousness proxies.

LRT is a statistical method based on calculation of likelihood ratio by using a comparative genomic data set for 32 vertebrates. Variants at conserved positions are considered as likely deleterious. LRT compares the probability of the data under a conserved model relative to a neutral model.

M-CAP is a pathogenicity likelihood score calculated with gradient boosting trees based on a number of features for sequences and their conservation in 99 primate, mammalian, and vertebrate genomes.

MetaSVM and MetaLR are metapredictors, i.e. combine predictions from other tools by using SVM and logistic regression (LR) algorithms, respectively. MutationAssessor uses evolutionary information coming from clustered MSAs of homologous sequences in subfamilies to analyze functional specificity on the background of conservation of overall function. Entropy function of the residue distribution is used as a measure of conservation and as an estimate for the impact of variants.

MutationTaster analyses evolutionary conservation, splice-site changes, loss of protein features and changes that might affect the amount of mRNA. MutPred utilizes SVM and RF methods for calculating the posterior probability that a residue has a certain structural or functional property and probability for the loss or gain of the property.

PolyPhen-2 uses eight sequence-based and three structure-based predictive features. The difference between HDIV and HVAR versions is the training sets. The former has been trained with Mendelian disease-causing variants annotated in the UniProt and affecting protein stability or function. HVAR version is trained on all the human disease-causing variants in UniProt, while SNVs without annotated involvement in disease are considered as benign. HDIV version is more suited to evaluate rare alleles at loci in complex phenotypes and HVAR in Mendelian diseases.

PON-P2 utilizes RF algorithm. Feature selection of 622 characteristics indicated that only 8 are essential for the predictor. PON-P2 utilizes information about evolutionary conservation, physical and biochemical properties of amino acids and GO annotations. PROVEAN uses an alignment score (*delta score)* as stability index. The larger the introduced difference to the score due to variation the more damaging is the variant. The tool collects a set of homologous proteins of the query protein and compute the delta score for each pairwise alignment.

REVEL is a RF-classifier based meta-predictor that uses results from MutPred, FATHMM, VEST, PolyPhen, SIFT, PROVEAN, MutationAssessor, MutationTaster, LRT, GERP, SiPhy, phyloP, and phastCons. The concept of SIFT is similar to REVEL. Evolutionary information is revealed from MSA and used to predict how tolerated the variant is. VEST is a RF-tool that prioritizes amino acid substitutions that alter protein activity.

### Scoring indices

In order to assess the performance of predictors we calculated six measures according to published guidelines [73, 74]. These measures are based on confusion/contingency matrix in which the actual conditions are compared with the prediction outcomes and data items are grouped as true positives (TP), true negatives (TN), false positives (FP) and false negatives (FN). Since the numbers of positive and negative cases in the datasets were not equal, the numbers were normalized to calculate the following measures.

The sensitivity or true positive rate (TPR) is the rate of TP over the total of positive conditions

$$ Sensitivity=\frac{TP}{TP+ FN}. $$

The specificity or true negative rate (TNR) is the rate of TP over the total of negative conditions

$$ Specificity=\frac{TN}{FP+ TN}. $$

The positive predictive value (PPV) or precision is the rate of true positive results over the total positive prediction

$$ PPV=\frac{TP}{TP+ FP}. $$

The negative predictive value (NPV) is the rate of true negative results over the total negative prediction:

$$ NPV=\frac{TN}{TN+ FN}. $$

The accuracy (ACC) is the rate of correctly predicted conditions over the total observations

$$ Accuracy=\frac{TP+ TN}{TP+ TN+ FP+ FN}. $$

The Matthews Correlation Coefficient (MCC) is a robust measure. It takes into account both over and under prediction and it returns a value from − 1 to + 1. + 1 identifies a perfect prediction, 0 identifies random prediction, and − 1 means a totally inverse prediction

$$ MCC=\frac{TP\times TN- FP\times FN}{\sqrt{\left( TP+ FP\right)\left( TP+ FN\right)\left( TN+ FP\right)\left( TN+ FN\right)}}. $$

The Overall Perfomance Measure (OPM) is a performance index used to visualize all the previous six indices simultaneously. OPM is represented by normalized volume of the performance cuboid, which ranges from 0 to 1 [30], where nMCC is calculated by rescaling the MCC value from 0 to 1:

$$ OPM=\frac{\left( PPV+ NPV\right)\left( Sensitivity+ Specificity\right)\left( Accuracy+\left(\frac{1+ MCC}{2}\right)\right)}{8}. $$

## Additional file

Additional file 1: **Table S1.** Numbers of proteins in membrane subcellular localizations. **Table S2.** Performance of subcellular localization predictors on MP1289 restricted to one subcellular localization per protein. **Table S3.** Performance of subcellular localization predictors on single and multi pass membrane proteins. (DOCX 21 kb)

## Data Availability

The datasets generated and analyzed during the current study are available in the VariBench repository [75].

## Abbreviations

ACC: accuracy
CAFA: Critical assessment of function annotation
CNN: Convolutional neural network
EC: Enzyme commission
ER: Endoplasmic reticulum
ExAC: Exome aggregation consortium
FFN: Feed forward network
FN: False negative
FP: False positive
GO: Gene ontology
GPI: Glycosylphosphatidylinositol
HMM: Hidden Markov model
HPA: Human protein atlas
HTP: Human transmembrane proteome
LSTM: Long-short-term memory
MCC: Matthews correlation coefficient
ML: Machine learning
MLP: Multi location protein
MP: Membrane protein
MP1289: Dataset for membrane proteins
MP508: Dataset for membrane proteins
mpHTP: Dataset for membrane proteins
NN: Neural network
NPV: Negative predictive value
OPM: Overall performance measure
PDB: Protein data bank
PPV: Positive predictive value
RF: Random forest
RNN: Recurrent neural network
SVM: Support vector machine
TM: Transmembrane
TMP: Transmembrane protein
TN: True negative
TNR: True negative rate
TP: True positive
TPR: True positive rate
