# Supplementary material for: Benchmarking subcellular localization and variant tolerance predictors on membrane proteins
Source: BMC Genomics. 2019 Jul 16;20(Suppl 8):547. doi: 10.1186/s12864-019-5865-0 (PMC6631444; doi:10.1186/s12864-019-5865-0)
Supplement: Supplementary file 1 — Table S1. Numbers of proteins in membrane subcellular localizations. Table S2. Performance of subcellular localization predictors on MP1289 restricted to one subcellular localization per protein. Table S3. Performance of subcellular localization predictors on single and multi pass membrane proteins. (DOCX 21 kb) [file 12864_2019_5865_MOESM1_ESM.docx]

**ProTstab – Predictor for cellular protein stability**

**Additional file**

Yang Yang^1,2,3^, Xuesong Ding^1^, Guanchen Zhu^1^, Abhishek Niroula^2^, Qiang Lv^1^ and Mauno Vihinen^2,*^

^1^ School of Computer Science and Technology, Soochow University, China

^2^ Department of Experimental Medical Science, BMC B13, Lund University, Lund, Sweden

^3^ Provincial Key Laboratory for Computer Information Processing Technology, Soochow University, China

Additional Table S1. Numbers of proteins in membrane subcellular localizations.

| Localization | MP1289 | | MP508 | |
| --- | --- | --- | --- | --- |
|  | No | % | No | % |
| Plasma membrane | 378 | 29.3 | 182 | 35.8 |
| Nuclear membrane | 84 | 6.5 | 32 | 6.3 |
| Cell Junctions | 80 | 6.2 | 37 | 7.3 |
| MLPs | 747 | 58 | 257 | 50.6 |

Additional Table S2. Performance of subcellular localization predictors on MP1289 restricted to one subcellular localization per protein.

| Method | BUSCA | CELLO | DeepLoc1.0 | LocTree3 | MultiLoc2 | SubCons | Wolf PSORT |
| --- | --- | --- | --- | --- | --- | --- | --- |
| TP | 149 | 166 | 253 | 183 | 58 | 124 | 263 |
| FP | 8 | 7 | 1 | 0 | 5 | 5 | 19 |
| TN | 403 | 486 | 387 | 537 | 496 | 301 | 523 |
| FN | 279 | 344 | 288 | 358 | 408 | 215 | 279 |
| Sensitivity | 0.35 | 0.33 | 0.47 | 0.34 | 0.12 | 0.37 | 0.49 |
| Specificity | 0.98 | 0.99 | 1.00 | 1.00 | 0.99 | 0.98 | 0.96 |
| PPV | 0.95 | 0.96 | 1.00 | 1.00 | 0.92 | 0.96 | 0.93 |
| NPV | 0.59 | 0.59 | 0.57 | 0.60 | 0.55 | 0.58 | 0.65 |
| ACC | 0.66 | 0.65 | 0.69 | 0.67 | 0.57 | 0.66 | 0.73 |
| MCC | 0.42 | 0.41 | 0.51 | 0.45 | 0.23 | 0.44 | 0.51 |
| OPM | 0.35 | 0.34 | 0.42 | 0.37 | 0.24 | 0.36 | 0.43 |

Additional Table S3. Performance of subcellular localization predictors on single and multi pass membrane proteins.

|  | BUSCA | | CELLO | | DeepLoc1.0 | | LocTree3 | | MultiLoc2 | | SubCons | | Wolf PSORT | |
| --- | --- | --- | --- | --- | --- | --- | --- | --- | --- | --- | --- | --- | --- | --- |
| Method | 1 TM | >1 TM | 1 TM | >1 TM | 1 TM | >1 TM | 1 TM | >1 TM | 1 TM | >1 TM | 1 TM | >1 TM | 1 TM | >1 TM |
| TP | 1307 | 2459 | 697 | 2404 | 1879 | 2363 | 1242 | 2635 | 325 | 862 | 871 | 1905 | 1442 | 2574 |
| FP | 104 | 111 | 75 | 76 | 115 | 130 | 75 | 111 | 49 | 66 | 47 | 64 | 113 | 146 |
| TN | 1848 | 2370 | 2228 | 2815 | 1920 | 2471 | 2290 | 2876 | 2174 | 2708 | 1683 | 2131 | 2284 | 2878 |
| FN | 633 | 151 | 1669 | 275 | 231 | 196 | 1117 | 364 | 1453 | 2014 | 787 | 492 | 950 | 448 |
| Sensitivity | 0.67 | 0.94 | 0.29 | 0.90 | 0.89 | 0.92 | 0.53 | 0.88 | 0.18 | 0.30 | 0.53 | 0.79 | 0.60 | 0.85 |
| Specificity | 0.95 | 0.96 | 0.97 | 0.97 | 0.94 | 0.95 | 0.97 | 0.96 | 0.98 | 0.98 | 0.97 | 0.97 | 0.95 | 0.95 |
| PPV | 0.93 | 0.96 | 0.90 | 0.97 | 0.94 | 0.95 | 0.94 | 0.96 | 0.87 | 0.93 | 0.95 | 0.97 | 0.93 | 0.95 |
| NPV | 0.74 | 0.94 | 0.57 | 0.91 | 0.89 | 0.93 | 0.67 | 0.89 | 0.60 | 0.57 | 0.68 | 0.81 | 0.71 | 0.87 |
| ACC | 0.81 | 0.95 | 0.63 | 0.94 | 0.92 | 0.94 | 0.75 | 0.92 | 0.62 | 0.63 | 0.75 | 0.88 | 0.78 | 0.90 |
| MCC | 0.65 | 0.90 | 0.35 | 0.88 | 0.83 | 0.87 | 0.55 | 0.84 | 0.27 | 0.37 | 0.56 | 0.77 | 0.59 | 0.81 |
| OPM | 0.55 | 0.85 | 0.30 | 0.82 | 0.77 | 0.82 | 0.46 | 0.78 | 0.27 | 0.32 | 0.47 | 0.69 | 0.50 | 0.74 |
